# Radiomics in precision medicine for colorectal cancer: a bibliometric analysis (2013–2023)

**DOI:** 10.3389/fonc.2024.1464104

**Published:** 2024-10-30

**Authors:** Hao Li, Yupei Zhuang, Weichen Yuan, Yutian Gu, Xinyan Dai, Muhan Li, Haibin Chen, Hongguang Zhou

**Affiliations:** ^1^ Department of Oncology, Affiliated Hospital of Nanjing University of Chinese Medicine, Nanjing, China; ^2^ Jiangsu Collaborative Innovation Center of Traditional Chinese Medicine Prevention and Treatment of Tumor, The First Clinical College of Nanjing University of Chinese Medicine, Nanjing, China; ^3^ Science and Technology Department, Jiangsu Collaborative Innovation Center of Traditional Chinese Medicine Prevention and Treatment of Tumor, Nanjing University of Chinese Medicine, Nanjing, China

**Keywords:** colorectal cancer, radiomics, bibliometric analysis, imaging biomarkers, precision medicine

## Abstract

**Background:**

The incidence and mortality of colorectal cancer (CRC) have been rising steadily. Early diagnosis and precise treatment are essential for improving patient survival outcomes. Over the past decade, the integration of artificial intelligence (AI) and medical imaging technologies has positioned radiomics as a critical area of research in the diagnosis, treatment, and prognosis of CRC.

**Methods:**

We conducted a comprehensive review of CRC-related radiomics literature published between 1 January 2013 and 31 December 2023 using the Web of Science Core Collection database. Bibliometric tools such as Bibliometrix, VOSviewer, and CiteSpace were employed to perform an in-depth bibliometric analysis.

**Results:**

Our search yielded 1,226 publications, revealing a consistent annual growth in CRC radiomics research, with a significant rise after 2019. China led in publication volume (406 papers), followed by the United States (263 papers), whereas the United States dominated in citation numbers. Notable institutions included General Electric, Harvard University, University of London, Maastricht University, and the Chinese Academy of Sciences. Prominent researchers in this field are Tian J from the Chinese Academy of Sciences, with the highest publication count, and Ganeshan B from the University of London, with the most citations. Journals leading in publication and citation counts are *Frontiers in Oncology and Radiology*. Keyword and citation analysis identified deep learning, texture analysis, rectal cancer, image analysis, and management as prevailing research themes. Additionally, recent trends indicate the growing importance of AI and multi-omics integration, with a focus on improving precision medicine applications in CRC. Emerging keywords such as deep learning and AI have shown rapid growth in citation bursts over the past 3 years, reflecting a shift toward more advanced technological applications.

**Conclusions:**

Radiomics plays a crucial role in the clinical management of CRC, providing valuable insights for precision medicine. It significantly contributes to predicting molecular biomarkers, assessing tumor aggressiveness, and monitoring treatment efficacy. Future research should prioritize advancing AI algorithms, enhancing multi-omics data integration, and further expanding radiomics applications in CRC precision medicine.

## 1 Introduction

Colorectal cancer (CRC) is one of the most prevalent malignant tumors globally and a leading cause of cancer-related mortality. In 2020, approximately 1.9 million new CRC cases and over 900,000 related deaths were reported globally (1). Projections indicate that, in 2040, these figures will rise to 3.2 million cases and 1.6 million deaths (2). Research indicates a positive correlation between CRC incidence and the Human Development Index, with higher rates observed in developed countries compared to those in developing countries (3). Developed countries have managed to control CRC morbidity and mortality somewhat effectively in recent years through early screening programs and the adoption of precision medicine strategies (4). Conversely, in developing countries, CRC incidence is escalating because of rapid economic and social transformations. The absence of robust healthcare systems and effective clinical management strategies presents a significant challenge in these regions (5, 6). Thus, it is crucial for developing countries to enhance healthcare investments, expand early screening programs, and advance precision medicine to mitigate the CRC burden.

Radiomics, a field of medical image analysis that blends multiple disciplines and omics technologies, was formally introduced by Dutch scholar Lambin P in 2012 (7). This field utilizes computer algorithms to extract and analyze quantitative features from conventional medical imaging data for applications including tumor prediction, screening, treatment planning, treatment response assessment, and prognosis (8). The workflow of radiomics typically involves image acquisition and preprocessing, image segmentation, feature extraction and selection, and model development and application (9). Quantitative image features, or image biomarkers, capture tumor tissue and lesion characteristics non-invasively and reflect the molecular biology of tumors, providing clinicians with comprehensive and objective reference indicators (10). An expert panel organized by Cancer Research UK and the European Organization for Research and Treatment of Cancer has proposed 14 key recommendations to standardize and promote the clinical translation of imaging biomarkers. These recommendations include widely used clinical oncology decision-making tools such as American College of Radiology Breast Imaging-Reporting and Data System (ACR BI-RADS) breast morphology, clinical Tumor, Node, Metastasis (TNM) stage, and others (11). Notably, the circumferential resection margin status has proven particularly valuable in assessing the prognostic value of patients with CRC, with high-resolution MRI providing superior assessment capabilities compared to American Joint Committee on Cancer (AJCC) TNM-based risk assessment criteria for local recurrence, disease-free survival, and overall survival (12). Furthermore, the integration of radiomics and radiogenomics holds the potential to replace traditional biopsy methods in predicting tumor characteristics and outcomes in CRC, offering new avenues for CRC screening, early diagnosis, and precision medicine (13).

Bibliometrics is a quantitative analysis technique that employs mathematics, statistics, and scientometrics to assess the key contributors—countries, institutions, and authors—and identify major research hotspots in various fields. This method also uncovers potential research patterns and forecasts future trends (14). Currently, it is extensively applied in oncology and radiology, enhancing the depth and breadth of research and providing robust data support for future studies. Aggarwal et al. (15) utilized bibliometric methods to analyze global lung cancer research trends between 2004 and 2013, identifying a focus on genetics, systemic therapy, and prognostic biomarkers while noting a decline in clinical translational research output. Their further analysis of publications on radiotherapy from 2001 to 2015 highlighted a disparity in the volume of research outputs and citations among countries, suggesting increased support for radiotherapy research in low- and middle-income countries (16). These findings underline the pivotal role of bibliometrics in identifying research frontiers and shaping future research directions. Since 2013, radiomics research in CRC has expanded to include preoperative diagnosis, clinical efficacy assessment, and prognosis prediction. However, the results have not been systematically reviewed, potentially leading to redundancy, unclear directions, and integration challenges in future studies. Although several studies employing bibliometrics to investigate radiomics in CRC provide valuable insights, more comprehensive analyses are needed to fully harness the literature available and enhance clinical applications and trend predictions. Furthermore, there is often a need for more detailed summaries of biomarkers to enrich these studies (17).

Utilizing a bibliometric approach, this study systematically evaluates the current status and future trends of radiomics applications in CRC precision medicine. By providing a comprehensive review of radiomics in the diagnosis and treatment of CRC, this study explores the relationship between research funding distribution and thematic trends, highlighting the application of imaging biomarkers, PET/CT, visualization techniques, and artificial intelligence (AI) in the field. This analysis offers insights for researchers to refine their future research strategies and to promote the further development and application of radiomics in CRC management.

## 2 Methods

### 2.1 Selection of databases

We selected the Web of Science Core Collection (WoSCC) from Clarivate Analytics as our primary literature source due to its rigorous indexing standards, which ensure the inclusion of high-quality literature. This selection strategy is aligned with Bradford’s Law, which asserts that the most significant scientific discoveries are predominantly published in core journals (18). This approach makes WoSCC an ideal tool for accurately tracking and analyzing key scientific outcomes, aiding in the identification of research hotspots and trends, thereby providing a solid data foundation for informed research decisions.

### 2.2 Definition of the time frame of the study

The study period was set from 1 January 2013 to 31 December 2023. This time frame was chosen because radiomics was formally introduced in 2012, and its application in CRC began gaining traction in 2013. Analyzing literature from this period allows for a comprehensive review of the field’s evolution from its initial stages to more recent developments. Additionally, using a full year’s data helps maintain continuity and clarity in the analysis of publication trends, avoiding potential errors from truncated data and revealing long-term developmental trends and pivotal moments in radiomics applied to CRC.

At the same time, we used Scopus data for cross-validation, which enhances the robustness of this study. Detailed analysis of the results can be found in the **Supplementary Materials**.

### 2.3 Literature search

**Figure 1:**
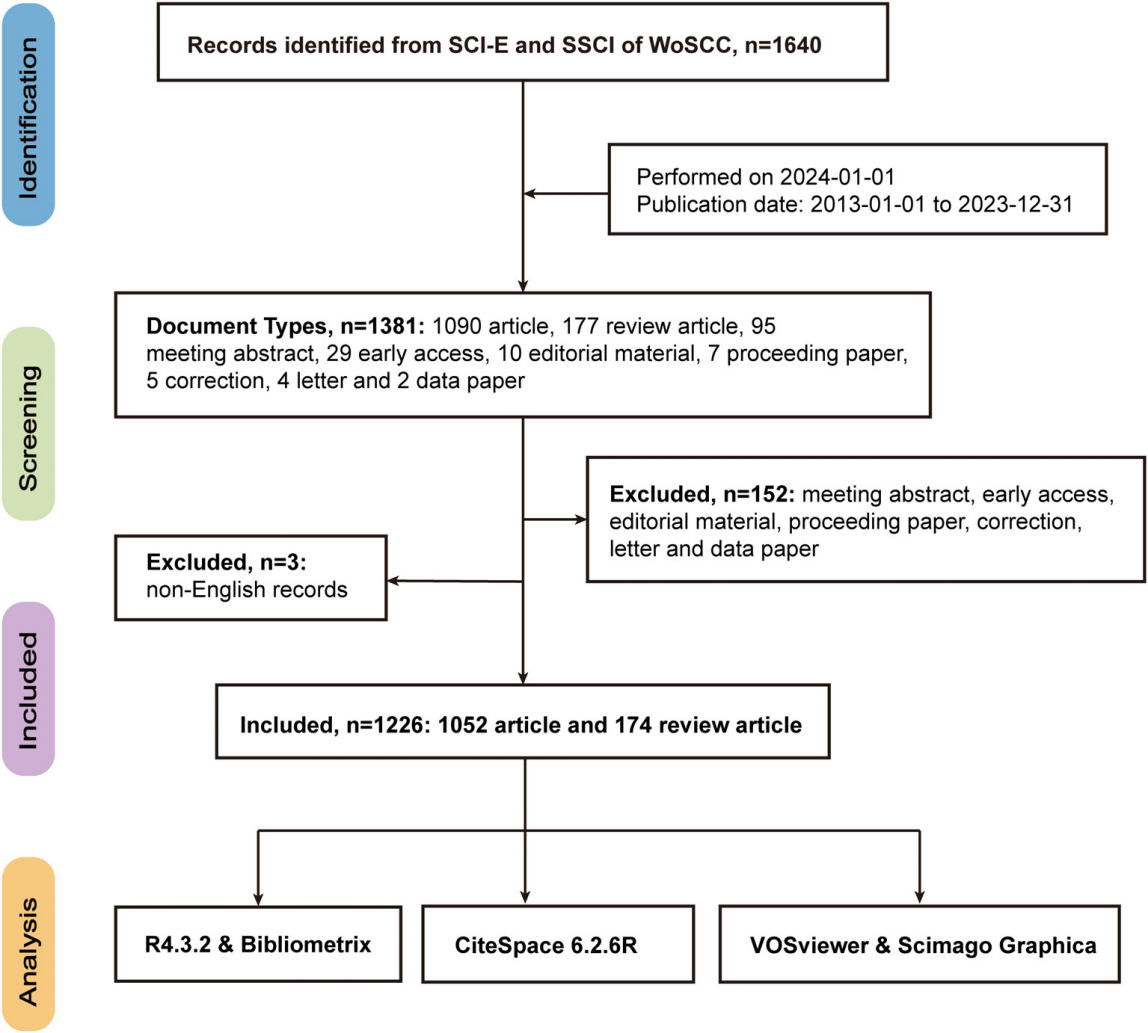
Flowchart of literature retrieval.

The literature search was conducted on 1 January 2024, targeting publications related to CRC and radiomics from 1 January 2013 to 31 December 2023. The search formula was TS = [(“Colorectal Neoplasm*” OR “Colorectal Tumor*” OR “Colorectal Cancer*” OR “Colorectal Carcinoma*” OR “Colonic Neoplasm*” OR “Colon Neoplasm*” OR “Colon Cancer*” OR “Colonic Cancer*” OR “Cancer of the Colon “ OR “Colon Adenocarcinoma*” OR “Rectal Neoplasm*” OR “Rectum Neoplasm*” OR “Rectal Tumor*” OR “Cancer of Rectum “ OR “Rectal Cancer*” OR “Rectum Cancer*”) AND (“radiomics” OR “image-based phenotyping” OR “imaging biomarkers” OR “texture analysis” OR “radiogenomics” OR “quantitative imaging” OR “imaging genomics” OR “image analysis” OR “computer-aided diagnosis” OR “machine learning in imaging”)]. Two researchers (Yupei Zhuang and Weichen Yuan) independently performed the searches to ensure the accuracy of the results. Inclusion criteria specified that the literature must be research articles or reviews published in English between 1 January 2013 and 31 December 2023. We followed the Preferred Reporting Items for Systematic Reviews and Meta-Analyses (PRISMA) guidelines in the selection of studies, where the inclusion and exclusion criteria were defined *a priori*, and the search strategy was documented to ensure reproducibility. The data were saved as “Full Record and Cited References” and exported as a plain text file. The search process is illustrated in **Figure 1**.

### 2.4 Data analysis tools

Various tools were employed for data analysis in this study. Adobe Illustrator 2022 was used to create a visual flowchart of the literature screening criteria and process. Microsoft Excel 2019 facilitated data cleaning and preliminary statistical analysis, such as frequency counting and trend plotting. For bibliometric analysis, we utilized the Bibliometrix package (19), VOSviewer (20), and CiteSpace (21).

Bibliometrix Analysis (version 4.1.3): To analyze the publication volume of authors and generate a timeline graph, follow these steps: Compress the exported.txt file into a.zip format. Initiate Bibliometrix 4.1.3 using R and sequentially navigate through “Import or Load Files” and “Import Raw File(s).” Choose “Web of Science (WoS/WoK).” Select the compressed.zip file. For data selection, choose “Authors” and “Country Scientific Production.”

VOSviewer Co-occurrence and Network Visualization (version 1.6.19): For co-occurrence analysis and temporal network visualization: Open VOSviewer and select “Create a map based on bibliographic data.” Opt for “Read data from bibliographic database files” and choose “Web of Science” for database selection. Import the pre-processed text files. Set the “Type of analysis” to “Co-authorship” and select “authors,” “organizations,” and “countries” as units of analysis. Use “Full counting” as the counting method. Adjust parameters for “network visualization” and “overlay visualization.” Utilize Scimago Graphica to geo-visualize the volume of national publications derived from VOSviewer outputs.

CiteSpace Cluster and Trend Analysis (version 6.2 R6): For keyword and reference cluster analysis, temporal analysis, and detection of emerging trends: Prepare the working directory with four new folders: “input,” “output,” “data,” and “project.” Save the downloaded file as “download_xx.txt” in the “input” folder. Process the file and initiate a new project in CiteSpace. For “Time Slicing,” select the desired analysis period. For “Node Types,” choose “Keyword” and “Reference,” and configure the “g-index” with k = 25. Depending on the analysis needs, select the algorithm “Pathfinder” and execute cluster analysis, temporal analysis, and detection of emerging trends. Generate overlay maps of journals using the “Overlay Maps” module.

Further descriptions of these tools and their operations can be found in the respective software manuals.

## 3 Results

### 3.1 Trends in the number of publications

**Figure 2:**
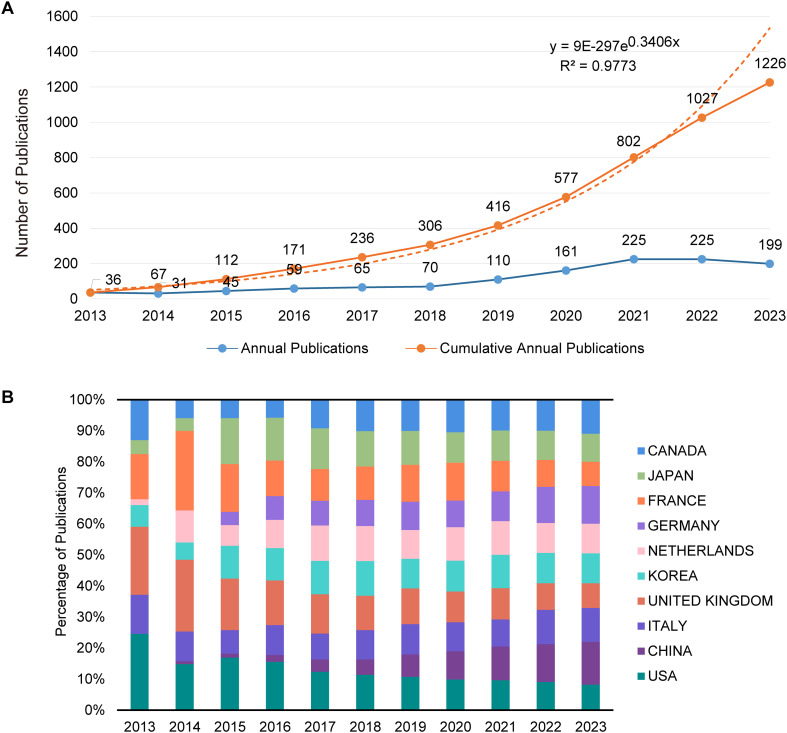
Analysis of publication trends and distribution. **(A)** Trends in annual and cumulative annual growth in publications: This graph displays two trends: the annual number of publications and the cumulative total over time from 2013 to 2023. The annual trend line illustrates the number of publications yearly, showing fluctuations and growth patterns. The cumulative trend line, overlaid on the same graph, reflects the total number of publications accruing over the specified period, providing a visual representation of overall growth in the field. **(B)** Percentage of annual publications in top 10 countries: This bar chart represents the distribution of publications by the top 10 contributing countries each year from 2013 to 2023. It highlights the percentage share of each country’s publications relative to the total annual output, showing shifts in the research landscape and indicating which countries are leading or increasing their contributions over time.

A total of 1,226 relevant publications from 2013 to 2023 were included in this analysis. Trends in the annual number of publications over this period are depicted in **Figure 2A**. From 2013 to 2018, there was a gradual increase in publication output, with an average annual count below 100. Starting in 2019, there was a notable rise in the number of publications, averaging over 180 annually. However, a slight decline was observed in 2022 and 2023. An exponential growth model applied to the cumulative publication data yielded a goodness-of-fit R² = 0.9773, demonstrating that the model accurately reflects the publication growth trend with high precision.

### 3.2 Country/regions and institutional analysis

A total of 1,869 research institutions across 69 countries/regions were included in this analysis. **Table 1** lists the top 10 countries/regions and institutions based on the number of publications. China leads with 406 publications, followed by the United States with 263, the United Kingdom with 126, and Italy with 133; each has generated over 100 publications. Whereas China has the highest publication count, the United States leads in terms of total citations. **Figure 2B** illustrates the distribution of publications by the main contributing countries/regions. In 2013, Canada, France, the United Kingdom, Italy, and the United States were prominent in publication numbers. Since 2015, the share of publications from countries like Germany and China has seen significant growth.

**Table 1:** Top 10 countries/regions and institutions by number of publications.

Country	Count	Citation	Institution	Count	Centrality
USA	263	11,524	General Electric	56	0.18
China	406	9,369	Harvard University	38	0.29
UK	126	5,342	University of London	38	0.2
Italy	133	3,308	Maastricht University	35	0.42
Germany	78	2,602	Chinese Academy of Sciences	32	0.12
Netherlands	66	2,275	SUN Yat-Sen University	30	0.13
France	48	2,205	Fudan University	30	0.24
Japan	61	1,934	Sichuan University	28	0.1
South Korea	70	1,623	Harvard Medical School	26	0.14
Switzerland	28	1,040	Massachusetts General Hospital	20	0

**Figure 3:**
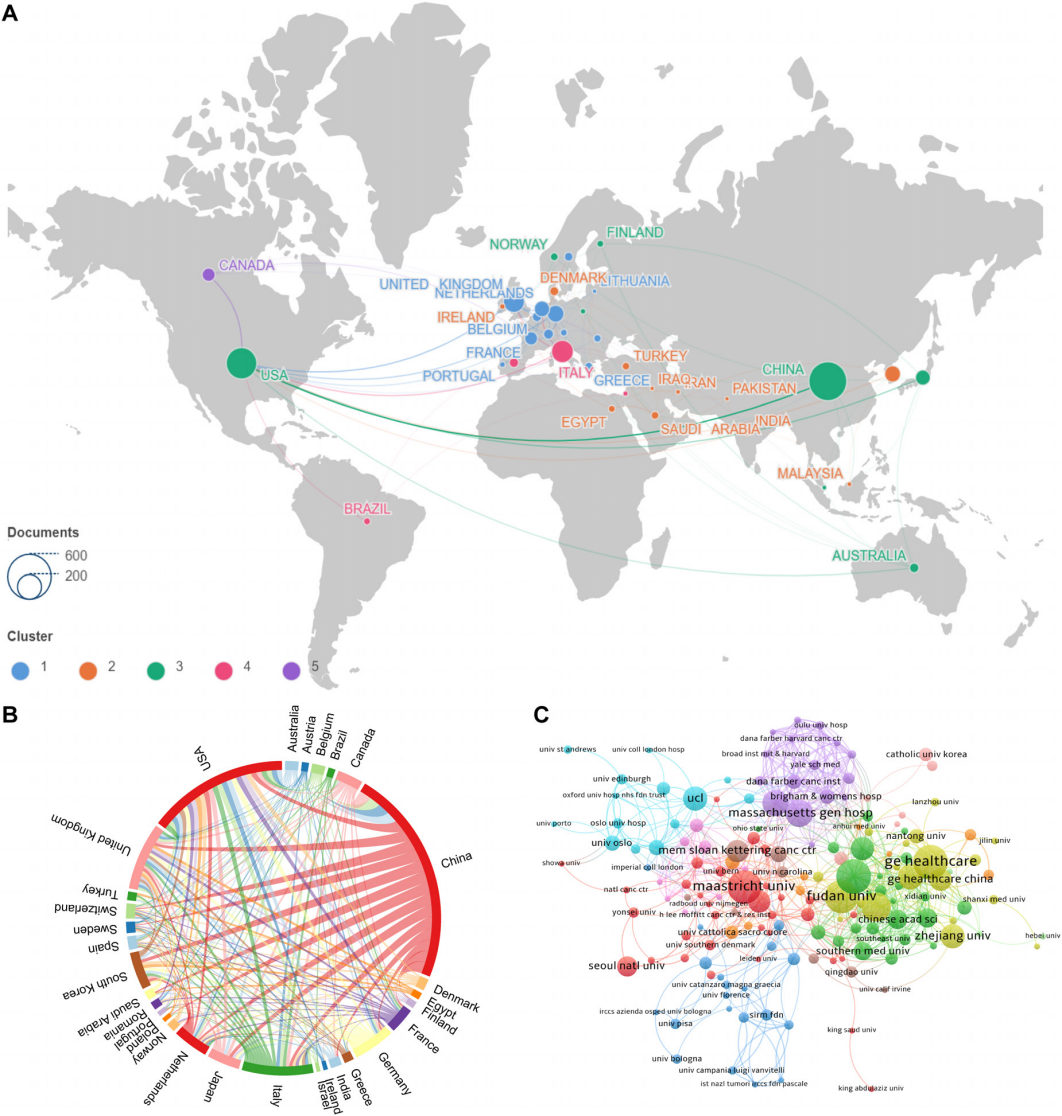
Visualizations of collaborative patterns in research. **(A)** Geographic visualization of country origination collaboration : This map illustrates the geographical distribution of collaboration among countries involved in the research. Countries are color-coded based on the intensity of their collaborative efforts, with warmer colors indicating higher levels of cooperation. **(B)** Country origination collaboration chord map : This chord diagram depicts the interconnections between countries, with lines connecting countries that have collaborated on research. The thickness of the lines represents the volume of collaborative publications, providing a visual representation of the strength of partnerships. **(C)** Institutional collaboration clustering map : This map clusters institutions based on the extent of their collaboration. Institutions within the same cluster are closely connected through joint research efforts, and different clusters are indicated by varying colors. This visualization helps identify which institutions are central to the network and how they are interconnected.

**Figures 3A, B** depict the patterns of collaboration and cooperative relationships among countries/regions through geographic distribution maps, cooperation chord diagrams, and cooperation networks, respectively. Countries such as the United States, China, France, Japan, and Australia demonstrate close cooperation, whereas European nations like the United Kingdom, Italy, Spain, New Zealand, and Switzerland also show strong connectivity. Additionally, **Figure 3C** presents the patterns of collaboration between different research institutions through cluster analysis, emphasizing the emergence of tightly-knit collaborative groups. The top three institutions by number of publications are General Electric (56 publications), Harvard University (38 publications), and the University of London (38 publications). Maastricht University holds a central position in the global collaboration network, with a centrality index of 0.42, followed by Harvard University (centrality = 0.29) and Fudan University (centrality = 0.24).

### 3.3 Analysis of authors and co-cited authors

A comprehensive analysis included the literature of 7,230 authors. **Table 2** highlights the top 10 most prolific and most cited authors, pinpointing the key contributors in the field and their academic impact. Tian J from the Institute of Automation, Chinese Academy of Sciences, emerged as the author with the highest number of publications. Meanwhile, Ganeshan B from University College London Hospitals accumulated the most citations.

**Table 2:** Top 10 most prolific and most cited authors.

Author	Count	H-index	Cited author	Citation	H-index
Tian J	16	80	Ganeshan B	298	26
Liu ZY	12	55	Lambin P	263	92
Song B	12	18	Horvat N	217	18
Tong	12	7	Gillies RJ	197	99
Liu Zaiyi	11	37	Liu ZY	183	55
Shen F	11	7	Huang YQ	174	15
Feng	10	23	Siegel RL	168	59
Liang CH	10	36	Maas M	167	43
Pang PP	10	20	Miles K	157	48
Duan SF	9	24	Jemal A	155	139

**Figure 4:**
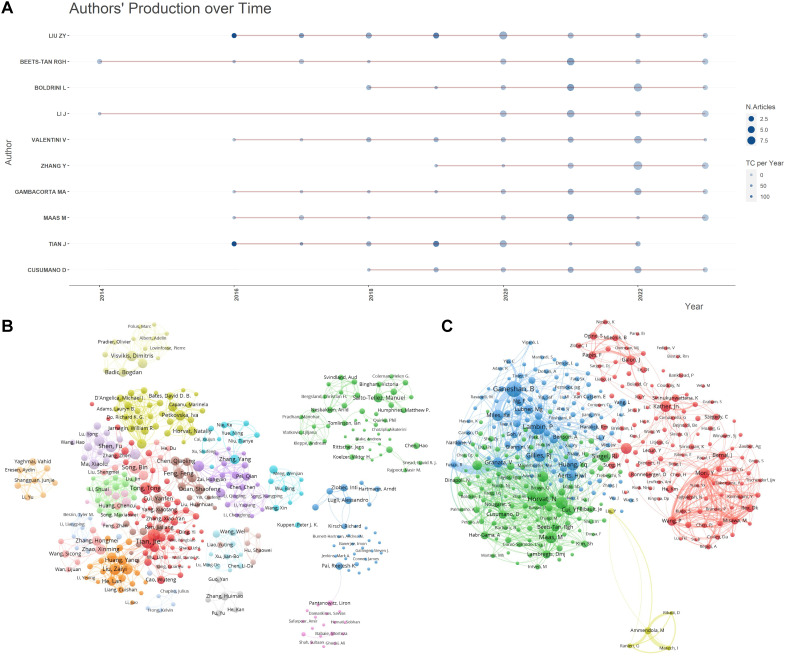
Author publication and collaboration visualizations. **(A)** Author publication timeline graph: This graph displays the publication timeline of key authors over the period from 2013 to 2023. It visualizes the volume of publications per year for each author, allowing for a comparative analysis of productivity over time. The graph aims to highlight trends in individual research outputs and identify periods of heightened activity. **(B)** Author collaboration clustering network graph : This network graph illustrates the collaboration patterns among authors, showing how researchers are interconnected through their joint works. The nodes represent individual authors, with node size indicating the number of publications. Links between nodes depict collaborative relationships and clusters within the network suggest groups of authors who frequently collaborate. This visualization helps identify key researchers and their networks within the community. **(C)** Co-cited author collaboration network graph : This graph maps the relationships among co-cited authors, showing which authors are frequently cited together in the literature. Nodes in this network represent authors, with lines between them indicating that their works are commonly cited in conjunction with one another. The positioning and proximity of nodes reflect the strength of co-citation links, highlighting influential authors whose work is foundational or pivotal in the field.

**Figure 4A** presents a line graph that tracks the publication output of these influential authors over time, illustrating changes in their research activity and highlighting periods of significant contributions. Liu ZY, also from the Institute of Automation, Chinese Academy of Sciences, is noteworthy for appearing in the top 10 for both publications and citations, indicating a consistent and substantial impact on the field since 2016. **Figure 4B** delineates the collaboration patterns and clustering relationships among the authors. In this network visualization, Tian J, Tomlinson I, Kirsch R, Gonen M, Pang P, Zhang Y, and Liu ZY stand out as central nodes linking various clustered groups, highlighting their roles in bridging different research communities. **Figure 4C** showcases the collaborative relationships among co-cited authors, with Jemal A, Siegel R, and Lambin P holding key positions within the co-citation network. This indicates their pivotal contributions as frequently referenced sources in the field, underscoring their influence on the research community.

### 3.4 Analysis of journals and co-cited journals

This analysis encompassed 369 academic journals, with **Table 3** presenting the top 10 journals based on the number of publications and citations. *Frontiers in Oncology* led with 78 publications, followed by *Cancers* with 65 and *Abdominal Radiology* with 41. Among these, eight journals are ranked in the JCR Q1 division and two in the Q2 division. Regarding impact factors, two journals boast an impact factor above 5, whereas the remaining eight have impact factors below this threshold. Notably, *Radiology*, *European Radiology*, *Journal of Clinical Oncology*, and *Scientific Reports* each accrued over 1,000 citations among the top cited journals.

**Table 3:** Top 10 journals by number of publications and citations.

Journal	Count	JCR (2023)	IF (2023)	Cited journal	Citation	JCR (2023)	IF (2023)
*Frontiers in Oncology*	78	Q2	3.5	*Radiology*	1,790	Q1	12.1
*Cancers*	65	Q1	4.5	*European Radiology*	1,544	Q1	4.7
*Abdominal Radiology*	41	Q2	2.3	*Journal of Clinical Oncology*	1,209	Q1	42.1
*European Radiology*	40	Q1	4.7	*Scientific Reports*	1,033	Q1	3.8
*World Journal of Gastroenterology*	31	Q1	4.3	*Clinical Cancer Research*	870	Q1	10.0
*Scientific Reports*	30	Q1	3.8	*Gastrointestinal Endoscopy*	778	Q1	6.7
*Academic Radiology*	23	Q1	3.8	*Plos One*	747	Q1	2.9
*Diagnostics*	23	Q1	3	*New England Journal of Medicine*	714	Q1	96.2
*European Journal of Radiology*	20	Q1	3.2	*Gastroenterology*	699	Q1	25.7
*Plos One*	15	Q1	2.9	*Radiologia Medica*	683	Q1	9.7

**Figure 5:**
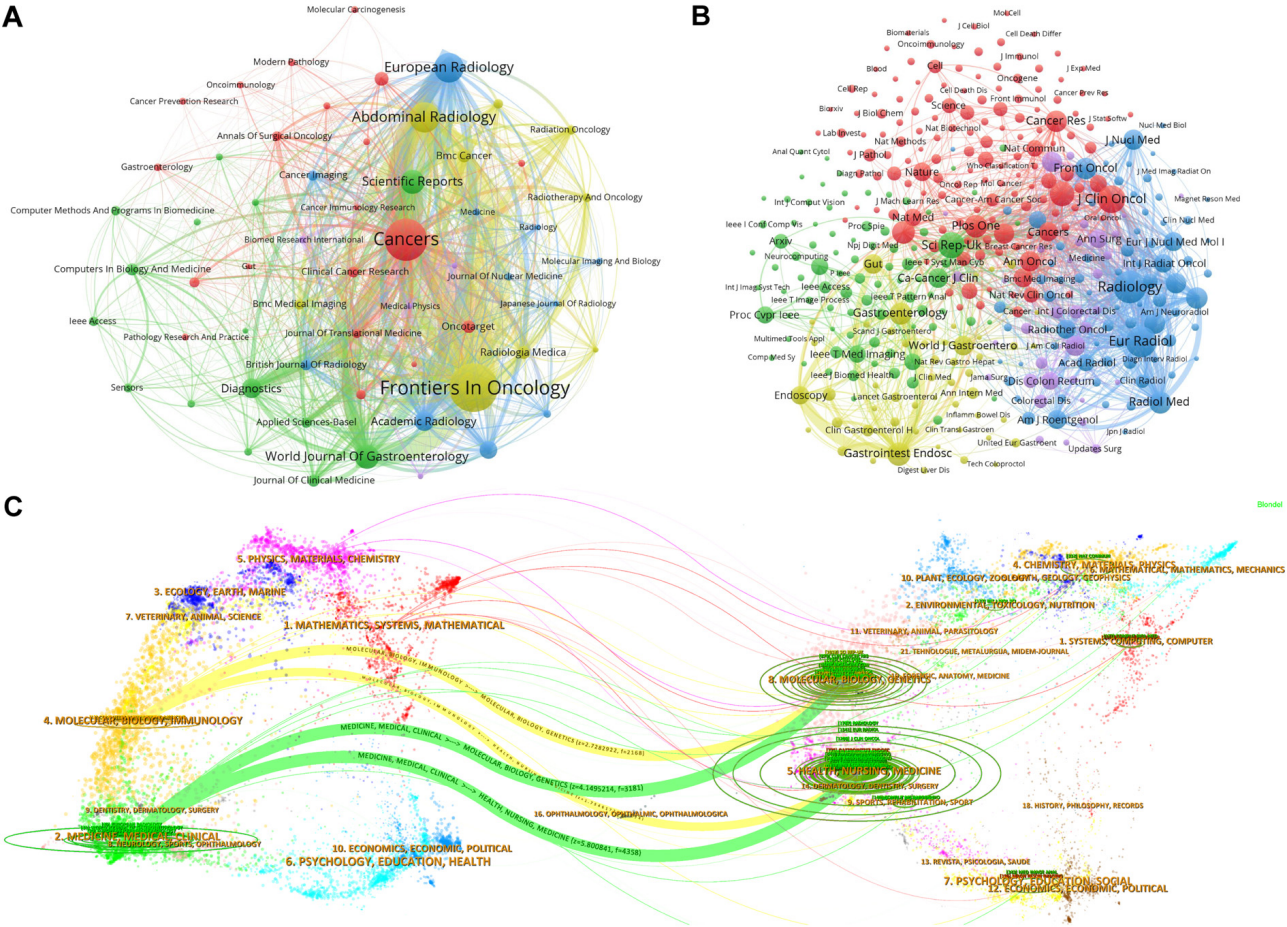
Visualization of journal collaborative and citation dynamics. **(A)** Journal collaborative clustering network: This graph displays a network visualization of journal collaborations, illustrating how journals within certain research fields or themes cluster together based on their joint publication efforts. The node size within the network represents the volume of collaborative outputs, and the thickness of the lines between nodes indicates the strength or frequency of collaborations. This clustering helps identify which journals frequently co-publish, suggesting areas of concentrated research activity and interdisciplinary connections. **(B)** Cited journal collaborative clustering network: This visualization focuses on the citation relationships among journals. Similar to the previous graph, nodes represent journals, but, here, the links depict citation relationships rather than co-authorship. The node size reflects the citation frequency, and connections between nodes show how often journals cite one another. This network provides insights into the influence and citation dynamics within the scholarly community, highlighting journals that serve as key references in various research domains. **(C)** Journal dual graph overlay: This dual graph overlay combines information about both the citing and cited journals, mapping the flow of information and influence across different disciplines and fields. The graph uses two layers: one representing journals that cite others (outgoing influence) and another for cited journals (incoming influence). The trajectory shown by a yellow curve illustrates significant trends in knowledge transfer between disciplines, emphasizing how certain fields are shaping research directions in others.

**Figure 5A** illustrates the collaborative network between journals through various clusters, where node size reflects the number of co-citations, and the line thickness indicates the closeness of collaboration. The prominent node in the red cluster underscores the central role of *Cancers* in facilitating inter-journal collaboration. **Figure 5B** identifies collaboration patterns between co-cited journals, with *Scientific Reports* and *Clinical Cancer Research* playing pivotal roles in fostering inter-journal collaboration. **Figure 5C**, a double plot overlay, shows the distribution of citing and cited journals, highlighting the direction of knowledge flow and interactions between disciplines. Cited journals are predominantly located within disciplines such as medicine and clinical science (Discipline 2#) and molecular biology and immunology (Discipline 4#), whereas citing journals mainly pertain to health, nursing, and medicine (Discipline 5#) and molecular biology and genetics (Discipline 8#). The yellow trajectory underscores the significant influence of publications from the medical and clinical fields, impacted by developments in health care and medicine (z = 5.80, f = 4,358) and molecular biology and genetics (z = 4.15, f = 3181). This visualizes the interdisciplinary research trends and the emergence of new fields.

### 3.5 Analysis of references

**Figure 6:**
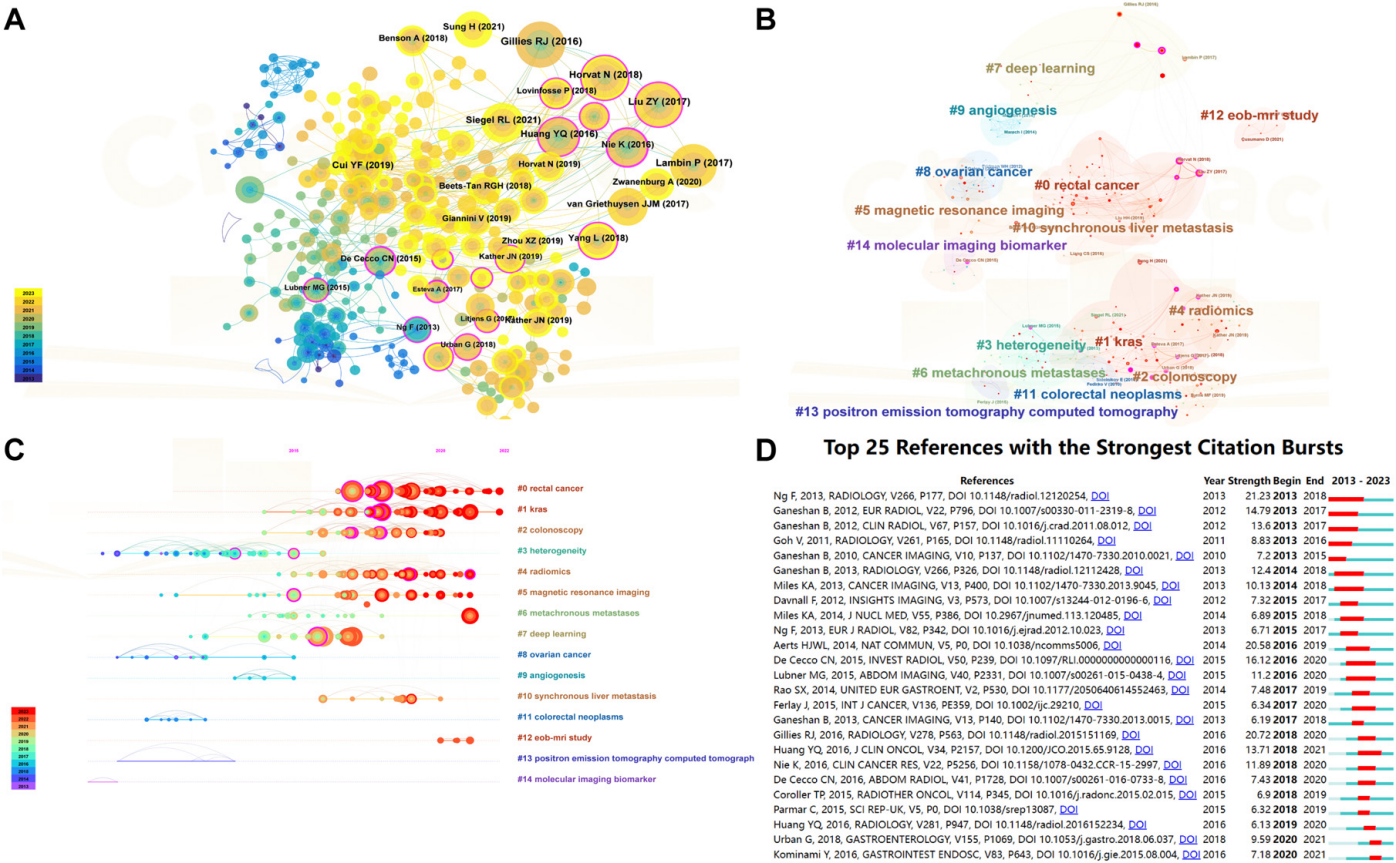
Visualization of reference co -citation and citation dynamics. **(A)** Reference co-citation visualization: This graph displays a network of how often references are cited together within the literature, illustrating the interconnectedness of studies based on shared citations. Nodes represent individual studies, and the lines between them indicate a co-citation relationship. The size of each node reflects the frequency of co-citations, highlighting references that commonly appear together in the literature and suggesting their thematic or methodological similarities. **(B)** Reference co-citation clustering visualization : This visualization segments the co-citation network into clusters, each representing a group of studies that are frequently cited together, suggesting they pertain to related topics or methodologies. Different colors are used to distinguish each cluster, aiding in the visual discrimination of thematic groups within the broader research landscape. **(C)** Timeline plot of reference co-citation clustering: This timeline graph plots the clusters of co-cited references over time, showing the evolution of research themes and the emergence of new trends. Each cluster is plotted against the year to demonstrate when certain topics gained prominence or faded in relevance, providing insights into the historical and developmental trajectory of research in the field. **(D)** Top 20 references with the highest citation explosion rate: This graph identifies the top 20 references that have experienced the most significant surges in citations over a specific period, indicating their rising influence or foundational impact on current research. Each reference is represented by a bar, the length of which corresponds to the “explosion” rate of citations, and color-coded to indicate the duration of the citation surge.

A comprehensive analysis incorporated 35,353 references cited across studies related to CRC. **Table 4** presents the 10 most cited references, highlighting their significance in the field. The article “Radiomics: Images Are More than Pictures, They Are Data” by Gillies et al. topped this list with 113 citations. This foundational study elaborated on the process, challenges, and potential of radiomics to enhance clinical decision-making, especially in oncology, setting a pivotal intellectual groundwork for further research on the application of radiomics in CRC (22). **Figure 6A** visualizes the citation dynamics of the referenced literature from 2021 to 2023. This timeline graph and node size variation reflect the rapid advancements and significant achievements in the field over recent years. The evolution of research hotspots is further delineated in **Figure 6B**, a co-citation clustering network graph, and **Figure 6C**, which offers a temporal view across 15 thematic categories. The top five themes identified were “#0 rectal cancer,” “#1 kras,” “#2 colonoscopy,” “#3 heterogeneity,” and “#4 radiomics.” **Figure 6D** displays the top 25 references with the highest citation bursts, using a red bold line to indicate the duration of each citation surge. The standout paper, “Assessment of Primary Colorectal Cancer Heterogeneity by Using Whole-Tumor Texture Analysis: Contrast-enhanced CT Texture as a Biomarker of 5-year Survival,” demonstrated a correlation between CT texture characteristics of primary CRC and 5-year overall survival, proposing that CT texture analysis might serve as a predictive biomarker for long-term survival in patients with CRC (23).

**Table 4:** Top 10 most cited references.

Title	DOI	First author	Year	Journal	Citation
Radiomics: Images Are More than Pictures, They Are Data	10.1148/radiol.2015151169	Gillies RJ	2016	*Radiology*	113
Radiomics Analysis for Evaluation of Pathological Complete Response to Neoadjuvant Chemoradiotherapy in Locally Advanced Rectal Cancer	10.1158/1078-0432.CCR-17-1038	Liu ZY	2017	*Clinical Cancer Research*	105
Radiomics: The Bridge between Medical Imaging and Personalized Medicine	10.1038/nrclinonc.2017.141	Lambin P	2017	*Nature Reviews Clinical Oncology*	103
MR Imaging of Rectal Cancer: Radiomics Analysis to Assess Treatment Response after Neoadjuvant Therapy	10.1148/radiol.2018172300	Horvat N	2018	*Radiology*	100
Cancer Statistics, 2021	10.3322/caac.21254	Siegel RL	2021	*CA: A Cancer Journal for Clinicians*	86
Development and Validation of a Radiomics Nomogram for Preoperative Prediction of Lymph Node Metastasis in Colorectal Cancer	10.1200/JCO.2015.65.9128	Huang YQ	2016	*Journal of Clinical Oncology*	76
Radiomics Analysis of Multiparametric MRI for Prediction of Pathological Complete Response to Neoadjuvant Chemoradiotherapy in Locally Advanced Rectal Cancer	10.1007/s00330-018-5683-9	Cui YF	2019	*European Radiology*	74
Rectal Cancer: Assessment of Neoadjuvant Chemoradiation Outcome Based on Radiomics of Multiparametric MRI	10.1158/1078-0432.CCR-15-2997	Nie K	2016	*Clinical Cancer Research*	73
Computational Radiomics System to Decode the Radiographic Phenotype	10.1158/0008-5472.CAN-17-0339	van Griethuysen JJM	2017	*Cancer Research*	73
Global Cancer Statistics 2020: GLOBOCAN Estimates of Incidence and Mortality Worldwide for 36 Cancers in 185 Countries	10.3322/caac.21660	Sung H	2021	*CA: A Cancer Journal for Clinicians*	67

### 3.6 Keyword analysis

**Figure 7:**
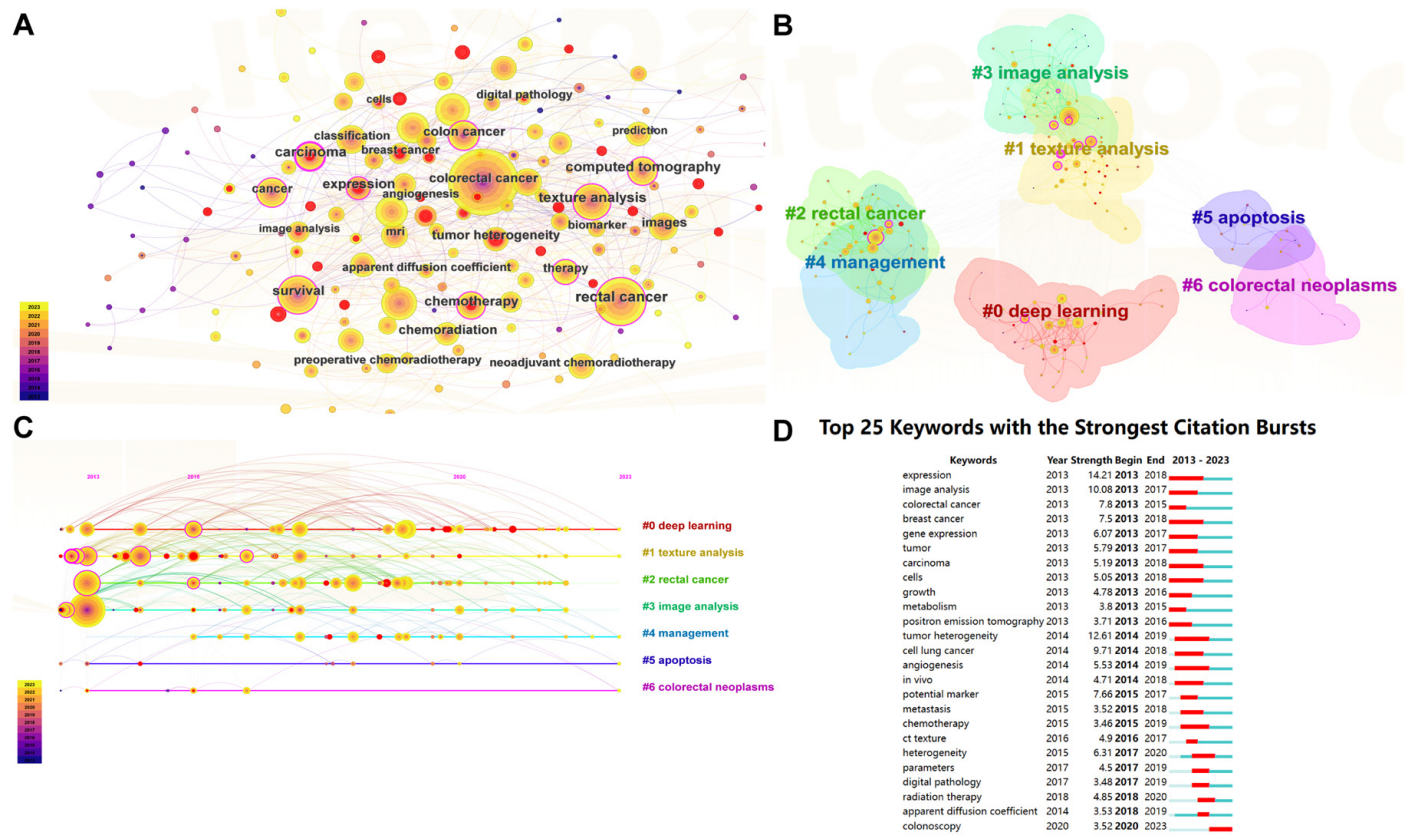
Visualizations of keyword dynamics and impact in research. **(A)** Keyword network visualization : This diagram illustrates the network of relationships among the most frequently used keywords within the field of CRC research. The graph displays keywords as nodes, with lines connecting them based on their co-occurrence in the literature. The size of each node indicates the frequency of the keyword”s appearance, providing insight into the prominence of certain topics. **(B)** Keyword clustering visualization: This graph segments the keyword network into clusters based on their associations, revealing groups of keywords that frequently appear together. Each cluster is color-coded to differentiate thematic groupings, making it easy to identify related research areas at a glance. **(C)** Keyword clustering timeline graph : This timeline graph depicts the emergence and evolution of keyword clusters over time. It helps trace the development of research themes within the field, showing when specific topics gained relevance or became less prominent. This dynamic view facilitates understanding of trends and shifts in the research focus over the years. **(D)** Top 25 burst words: This chart identifies keywords that have experienced significant bursts in usage within a defined period, indicating emerging trends or sudden increases in research activity around specific topics. Each keyword is represented by a bar, the length of which corresponds to the intensity of the burst, and is color-coded to illustrate the duration of the surge in popularity.

Keywords are crucial in literature search and defining research domains as they encapsulate the core themes of scholarly work. **Figure 7A** presents a keyword network visualization, highlighting the top 10 keywords by frequency within the field. These include “colorectal cancer” (n = 493), “rectal cancer” (n = 252), “survival” (n = 173), “texture analysis” (n = 161), “artificial intelligence” (n = 122), “magnetic resonance imaging” (n = 120), “colon cancer” (n = 115), “deep learning” (n = 114), “classification” (n = 111), and “computed tomography” (n = 106). This visualization provides a graphical representation of the frequency and centrality of terms within the research landscape. **Figure 7B** explores the interconnectivity among these keywords through a clustering graph. This graph categorizes the keywords into distinct clusters based on their association with the literature, illustrating how different research topics are interlinked. **Figure 7C**, a timeline view of keyword clustering, tracks the evolution of these research themes over time. This dynamic representation helps identify how certain topics have gained or waned in prominence, reflecting shifts in research focus and technological advancements. The seven major research theme clusters identified are “#0 deep learning,” “#1 texture analysis,” “#2 rectal cancer,” “#3 image analysis,” “#4 management,” “#5 apoptosis,” and “#6 colorectal neoplasms.” **Figure 7D** highlights the top 25 keywords with the highest outbreak rate, indicating sudden increases in usage over a specific period. Notably, “expression,” “tumor heterogeneity,” and “image analysis” each show an outbreak intensity of more than 10, underscoring their emerging significance in recent research.

## 4 Discussion

In this study, we conducted a bibliometric analysis and knowledge graph visualization of 1,226 publications in the field of radiomics and CRC in the WoSCC from 2013 to 2023. This analysis aimed to visualize the publication trends, collaborative networks, research hotspots, and future directions in this field over the past 11 years.

### 4.1 General situation analysis

The analysis of annual publication trends reveals that the field’s development can be divided into three distinct phases: the early phase (2013–2018), the development phase (2019–2021), and the late phase (2022–2023). During the early phase, the publication growth was modest, primarily originating from countries like the United States, the United Kingdom, France, Canada, and Italy. These nations demonstrated early contributions to and advanced levels of radiomics, reflecting their longstanding intellectual engagement in oncology. The development phase saw a rapid increase in publications, with countries such as China, Germany, Japan, South Korea, and The Netherlands intensifying their contributions. This surge indicates a growing recognition of the field’s importance and an increase in resource allocation, which likely corresponds to the rising social and healthcare burdens associated with CRC. In the late phase, there was a decline in publication numbers, potentially due to the scarcity of high-quality multicenter studies, which poses challenges in translating imaging biomarkers from this field into clinical practice effectively. The top 10 institutions by publication volume include four each from China and the United States, with one each from the United Kingdom and The Netherlands. China leads in total publication count, likely driven by the recent escalation in CRC incidence linked to rapid economic changes, coupled with increased national investments in research resources. The United States, holding the highest total citation count, indicates the high impact and global recognition of its research outputs. Furthermore, it has been particularly active in fostering international collaborations; establishing networks with countries such as China, Japan, and Australia; and maintaining close ties with European nations like the United Kingdom, Italy, and Switzerland. This pattern suggests that future research should place greater emphasis on international cooperation to enhance research quality, facilitate the sharing of findings, and propel the field’s development.

Tian J (H-index = 80), from the Chinese Academy of Sciences, stands as the most prolific author in our analysis, with a total of 16 publications. His extensive research spans radiology, nuclear medicine, and medical imaging, earning him recognition as a cross-disciplinary Highly Cited Scholar for three consecutive years (2021–2023). This accolade underscores his significant contributions to the fields of radiology and nuclear medicine. In 2016, Tian J and his team developed an innovative radiomics model that integrates imaging features, CT-reported lymph node status, and clinical risk factors (24). This model serves as a crucial tool for the preoperative prediction of CRC lymph node metastasis and guides personalized treatment approaches. On the other hand, Ganeshan B (H-index = 26), from University College London, has amassed the most citations in this study, totaling 298. His pioneering work in 2013 on applying CT texture analysis to quantify tumor heterogeneity has been pivotal, providing essential insights for tumor imaging, treatment response assessment, and prognosis (25). The contributions of both have significantly advanced the use of radiomics in oncology, particularly in CRC, inspiring new directions in tumor diagnosis and precision medicine.

We analyzed the influence of funding on research trends by integrating information on national policies and the activity of research institutions. The pronounced research output and high citation rates in the United States and China underscore substantial investments in radiomics and oncology, aligning with these nations’ governmental priorities on medical technology and innovation. Significant funding initiatives such as the Information Technology Initiative for Cancer Research sponsored by the National Cancer Institute in the United States and the Natural Science Foundation of China in China are pivotal in driving vigorous research activities in these fields (26, 27). These programs underscore the commitment of both countries to advancing medical research and technology. In the realm of tumor radiomics, there is a discernible preference for developing specific research themes such as deep learning and multiparametric imaging models. This focus reflects the funding bodies’ support for projects with high potential for clinical translation, emphasizing emerging technologies and advanced data analysis techniques. This strategic funding has not only accelerated technological innovations but also fostered conditions conducive to significant scientific breakthroughs (28). Further analysis indicates that funding support is often channeled toward research institutions that participate in international collaborations. These collaborations are instrumental in expanding sample sizes and enhancing data diversity, thereby increasing the research’s impact and applicability (29). Given these insights, it is advisable for future funding strategies to prioritize clinically relevant research projects, especially those involving multicenter and multinational studies. Such a focus will likely enhance the clinical application of radiomics technologies and promote international academic exchange.

*Frontiers in Oncology* (IF = 3.5) leads as the journal with the most publications in this study. It encompasses all domains of cancer research, striving to enhance our understanding of cancer to refine diagnostic, therapeutic, and management strategies. The rising potential of radiomics in clinical management strategies for CRC has notably piqued the journal’s interest, steering its focus toward this innovative area. Additionally, many high-output journals are Open Access (OA). OA journals provide unrestricted access to their content, which can lead to a higher number of submissions as authors seek wider dissemination and increased citations of their work. The OA model facilitates academic communication and boosts the visibility and impact of research articles. *Radiology* (IF = 12.1) stands as the most cited journal. This journal is a cornerstone in the field of radiology and imaging, publishing works that command high authority in the realm of radiomics. Its contributions are instrumental in shaping the foundational and advanced understanding of radiomics applications. Among the top 10 most cited references, two discuss theoretical frameworks underlying the origins and prospects of radiomics. Two others are epidemiological studies analyzing global cancer trends, and six involve developmental trials of multiparametric radiomics models specifically targeting CRC. Collectively, these references underscore the substantial promise of radiomics in the assessment, prediction, and personalized treatment approaches for CRC, reflecting widespread global interest and vigorous research efforts in this arena. In terms of temporal milestones, the year 2015 marked a pivotal shift in research orientation. Before this year, studies predominantly focused on cancers such as non–small- cell lung cancer (30), metastatic renal cell carcinoma (31), and CRC (23), with imaging techniques mainly confined to CT texture analysis as a predictive biomarker. This period characterized the exploratory phase of radiomics in oncology (32). Post-2015, the research emphasis transitioned toward embracing novel technologies and addressing clinical challenges, including the application of deep learning, MRI in the context of rectal cancer (33), and studies on liver metastases (34). This shift indicates a robust movement toward precision medicine and personalized treatment strategies. Concurrently, the introduction and integration of advanced AI algorithms have magnified the significance of radiomics in cancer diagnosis and treatment, heralding a new era of innovation in medical imaging.

The keywords such as survival, texture analysis, and artificial intelligence stand out because of their high co-occurrence intensity, highlighting central research focuses and trends within the field. Survival emerges as a pivotal indicator of treatment outcome assessment, underscoring the research emphasis on the efficacy of various treatment modalities, including radiotherapy, chemotherapy, and neoadjuvant therapy. This focus suggests that enhancing patient survival remains a fundamental objective in CRC research and treatment strategies. Texture analysis, a cornerstone technology in radiomics, is instrumental in diagnosing, typing, and assessing the prognosis of diseases by analyzing textural features within medical images. Related keywords such as angiogenesis, tumor heterogeneity, and tumor classification further illuminate the scope of texture analysis in elucidating tumor biology and assessing treatment responses. The evolution of AI plays a critical role in advancing radiomics, particularly through the adoption of deep learning and convolutional neural network (CNN) technologies. These sophisticated algorithms have shown substantial benefits in processing multidimensional image data, automating feature extraction, and addressing complex multiparametric challenges. Although initial studies primarily utilized machine learning algorithms such as random forests, decision trees, and regression models, current research trends increasingly leverage more advanced AI techniques. This shift aims to amplify the contribution of radiomics to clinical decision-making, enhancing its utility and precision in the medical field (35).

In this study, IF and citation counts served as preliminary indicators of literature quality. Comparative analysis identified several issues in publications of lower quality: non-randomized and outdated research designs lacking theoretically supported hypotheses; data handling flaws, including insufficient sample sizes, opaque data processing, and reliance on single-center data; and problems related to clinical translation and practical application, characterized by high repetitiveness and low clinical application rates. The prevalence of such lower-quality literature may be attributed to academic bandwagoning and specific national policies. These factors have a limited contribution to defining the field’s central themes and trends and cannot be entirely mitigated by IF and citation counts, potentially introducing bias into our analysis of future trends. To address these shortcomings, we integrated bibliometrics with a systematic review to summarize recent research hotspots and project future trends. Furthermore, to enhance the quality of future research, we recommend that researchers adopt rigorous standards and appropriate statistical analysis methods in their study designs. It is also advised that researchers provide comprehensive details of their data collection and analysis to support independent verification and validation. Studies that are multicenter and interdisciplinary tend to improve research quality more effectively and are more likely to achieve clinically valuable outcomes.

### 4.2 Application of radiomics in CRC

Through comprehensive analysis of citations and keywords, seven major research theme clusters have been identified, showcasing the diversity of research hotspots within the field. These clusters span from biomarker discovery and technological advancements to clinical management strategies for CRC. This section summarizes and discusses the broad applications of radiomics in CRC management, emphasizing the prediction of molecular biomarkers, analysis of tumor aggressiveness, and assessment of treatment responses.

#### 4.2.1 Molecular biomarker prediction for CRC

The National Comprehensive Cancer Network recognizes high-frequency microsatellite instability (MSI-H), along with Kirsten rat sarcoma viral oncogene homolog (*KRAS*) and B-Raf proto-oncogene, serine/threonine kinase (*BRAF*) mutations, as clinically significant molecular markers of CRC (36). The identification of these biomarkers plays a crucial role in predicting disease progression and response to treatment. It also enables the provision of more precise and personalized treatment strategies for patients.

##### 4.2.1.1 MSI-H

In CRC, MSI-H serves as a sensitive indicator of defective DNA mismatch repair in approximately 15% of cases (37). Patients with MSI-H CRC typically show poor responses to conventional chemotherapy but exhibit favorable responses to immune checkpoint inhibitor therapy (38). Advanced radiomics models, particularly those incorporating machine learning, have proven effective in predicting MSI status, thus optimizing treatment strategies. For example, radiomics column line plots that combine clinical indicators with imaging features have demonstrated the ability to distinguish non–MSI-H from MSI-H cases effectively, with areas under the curve (AUC) ranging from 0.74 to 0.77 (39). The radiomics IROI3 model, which assesses a 3-mm contraction of the tumor’s largest area, non-invasively reflects intratumor heterogeneity and genetic instability, achieving an AUC of 0.908 (40). These models have also identified clinical factors such as older age, right-sided colon cancers, hypertension, and N-staging as independently associated with MSI-H. Additionally, a machine learning model that integrates Fluorodeoxyglucose (18F) (18F-FDG) PET/CT data with PET features and clinical parameters efficiently predicts MSI-H, offering a novel approach to personalizing treatment for patients with MSI-H CRC (41).

##### 4.2.1.2 *KRAS* and *BRAF* mutations

The *KRAS* and *BRAF* genes are crucial in regulating the epidermal growth factor receptor (EGFR) signaling pathway, particularly in the treatment of metastatic CRC (mCRC). Mutations in these genes often result in resistance and poor prognosis in response to anti-EGFR therapies, such as panitumumab and cetuximab (42, 43). Radiomics models, especially those using multiphase CT and deep learning techniques, have shown potential in predicting these mutations, thereby informing personalized therapy strategies. Hu et al. (44) found that multiphase CT radiomics models can effectively predict *KRAS* mutations, highlighting the importance of log-sigma-glrlm-LongRunEmphasis features associated with these mutations. The introduction of deep learning has further enhanced the accuracy and non-invasiveness of predicting *KRAS* mutations using CT imaging models (45). Similarly, Cui et al. (46) developed a T2-weighted imaging–based model that demonstrated moderate performance in predicting *KRAS* mutation status in rectal cancer patients, noting that an increased tumor axial-to-longitudinal size ratio correlates with a higher risk of *KRAS* mutations. A two-center study highlighted that *BRAF* mutations might lead to shorter survival in patients with CRC through radiomics modeling, with wavelet filtering features identified as optimal for predicting *BRAF* mutations (47). Moreover, texture features have been positively correlated with *BRAF* mutations and 5-year overall survival in patients with advanced CRC (48). These findings underscore the potential of radiomics in predicting *KRAS* and *BRAF* mutations in CRC, providing crucial insights for personalized patient treatment and guiding future research toward enhancing model performance and validating the clinical utility of these biomarkers.

#### 4.2.2 Analysis of CRC aggressiveness

The invasive nature of CRC is a crucial factor in tumor biology, directly influencing the prognosis of CRC and the choice of therapeutic strategies. Lymph node metastasis and neuroinvasion are key indicators for assessing tumor behavior (49, 50).

##### 4.2.2.1 Lymph node metastasis

Accurate preoperative prediction of lymph node metastasis is crucial for formulating treatment plans for CRC. It informs the extent of surgery and the necessity for adjuvant therapy (51). Huang et al. (24) developed a radiomics line chart that accurately predicted lymph node metastasis risk in patients with CRC by analyzing CT images, effectively stratifying patients. This highlights the potential of radiomics in detecting early metastasis, essential for preoperative planning. Moreover, deep learning has expanded the capabilities in this field by analyzing complex tumor features, thus enhancing clinical decision-making. Li et al. (52) utilized deep learning to integrate tumor and lymph node characteristics, improving risk stratification and prognostic accuracy for patients with stage II CRC. Further, the combination of deep learning and genomics has advanced imaging genomics, as shown by Zhao et al., who demonstrated that deep learning–selected features correlated with gene enrichment related to metabolic and immune pathways, significantly improving lymph node metastasis prediction (53). These advancements underscore the potential of radiomics to refine lymph node metastasis forecasts and preoperative planning in CRC, emphasizing the value of technology integration and interdisciplinary collaboration for comprehensive and personalized patient management.

##### 4.2.2.2 Perineural infiltration

Perineural infiltration (PNI) is detected in approximately 20% to 30% of surgically resected CRC samples. Its presence indicates a highly invasive tumor, associated with increased recurrence and decreased survival rates (54, 55). Integrating clinical markers with imaging features, radiomics accurately assesses PNI risk in patients with CRC, facilitating the development of tailored adjuvant treatment strategies (56). The use of 18F-FDG PET has proven effective in evaluating PNI risk. A specific radiomics model, utilizing 18F-FDG PET/CT data, has shown high accuracy in predicting PNI in patients with non-metastatic CRC. Independent predictors identified include Carcinoembryonic Antigen (CEA) levels, lymph node metastasis detected by PET/CT, and total lesion glycolysis (57). Additionally, MRI-based radiomics studies suggest that models integrating multiple imaging features outperform those based on a single feature. This advancement underscores the potential of radiomics to enhance CRC prognostic assessments, potentially supplanting traditional clinical prognostic factors such as depth of infiltration and offering novel insights for treatment strategies (58).

##### 4.2.2.3 Distant metastases

In the analysis of CRC aggressiveness, distant metastasis, particularly to the liver, is a definitive indicator of high tumor aggressiveness and spread. Consequently, early detection of liver metastases is critically important for effective patient management (59, 60). Recently, the integration of CT radiomics with AI algorithms, specifically CNNs, has emerged as a novel approach for the early diagnosis of CRC liver metastases. Studies have shown that radiomics models incorporating CNNs not only match the performance of radiologists in detecting small, low-attenuation liver nodules but also demonstrate higher diagnostic confidence (61). However, although radiomics models incorporate machine learning mirror clinical parameters in reflecting patient status, they do not provide additional prognostic benefits (62). This indicates that CNNs are superior to traditional machine learning techniques in enhancing the clinical utility of imaging histology. The application of radiomics in diagnosing CRC liver metastases and informing treatment decisions underscores its potential. Future research should focus on integrating data from various imaging modalities and incorporating clinical, pathological, and molecular biomarker information to enhance the predictive capabilities of these models and provide more accurate guidance for the early diagnosis and treatment of distant CRC metastases.

#### 4.2.3 Assessment of treatment response in CRC

CRC treatment employs various methods such as surgery, chemotherapy, radiotherapy, targeted therapy, and immunotherapy. Central to these treatments is the regular monitoring of treatment responses, which is essential for modifying treatment plans, enhancing efficacy, and ensuring patient quality of life. Radiomics plays a pivotal role in providing precise tumor and treatment response data, thereby optimizing personalized treatment strategies. Particularly when integrated with genomic data, radiomics has proven exceptionally valuable in assessing responses of patients with CRC to immune and targeted therapies. For instance, Huang et al. (63) demonstrated that, using an imaging genomics model combined with genomic analysis, several immune-related genes such as Platelet Endothelial Cell Adhesion Molecule 1 (*PECAM1*), PR Domain Zinc Finger Protein 1 (*PRDM1*), Allograft Inflammatory Factor 1 (*AIF1*), Interleukin 10 (*IL10*), Interferon Stimulated Exonuclease Gene 20 (*ISG20*), and Toll-Like Receptor 8 (*TLR8*) have a strong positive correlation with the imaging features of stage III CRC. This finding highlights the potential of these genes as personalized therapeutic targets. Additionally, texture analysis and radiomics parameters like SHAPE Volume, HISTO Kurtosis, and Gray-Level Run-Length Matrix (GLRLM) Gray-Level Non-Uniformity (GLNU) have shown promise in predicting responses to targeted therapies, aiding clinical decision-making (64). Further research has indicated the importance of the Notch-Jagged1 signaling pathway in prognosticating outcomes for patients with advanced CRC treated with bevacizumab; high marker expression correlates with early disease progression during treatment (65). Moreover, imaging histology, when combined with machine learning techniques like the RF5 model, has surpassed traditional methods in accurately predicting responses to adjuvant chemotherapy in patients with CRC (66). Radiomics is also effective in forecasting responses to chemotherapy and radiofrequency ablation in patients with distant metastatic CRC (67, 68). In conclusion, the interdisciplinary application of radiomics has significantly enhanced the accuracy of treatment response assessments and provided a foundation for personalized treatment decisions, showcasing its vast potential in CRC management.

### 4.3 Application of visualization techniques : PET/CT as an example

Visualization techniques play a central role in radiomics research. By transforming complex datasets into intuitive images or graphs, these tools not only facilitate a better understanding of imaging data but also reveal patterns and trends, especially in the analysis of tumor heterogeneity, morphological changes, and their correlation with patient prognosis. Additionally, visualization techniques enable the integration of diverse data sources, such as imaging data combined with genetic, protein, and other clinical information, providing a foundation for comprehensive disease mechanism analysis. This multidimensional data integration help overcome the limitations of single-source data, allowing for a deeper understanding of diseases and supporting more precise clinical decision-making.

PET/CT imaging is a prime example of the application of visualization techniques in radiomics, particularly in cancer treatment. For instance, studies have shown that 18F-FDG-PET and PET/CT are widely used in the treatment planning of patients with colorectal liver metastases, especially for selective internal radiation therapy with yttrium-90 (69). These imaging technologies provide more precise information on metabolic activity through visualization, allowing for better assessment of treatment response. Additionally, PET/CT is used in radiotherapy planning to define the biological target volume, combining metabolic and anatomical information to help physicians develop more accurate treatment plans (70). With these visualization tools, physicians can better identify and differentiate tumor heterogeneity, applying this knowledge to personalized treatment.

As radiomics continues to evolve, the integration of PET/CT data with other imaging modalities (such as MRI) and multi-omics data (such as genomic information) is becoming increasingly mainstream. This data fusion supports visualization techniques by offering additional dimensions of analysis, enabling a more comprehensive understanding of tumor biology. In this process, AI-driven visualization tools are also playing an important role, not only automating the analysis of complex images but also enhancing the ability to process large-scale datasets. These advancements are driving the application of radiomics in precision medicine, particularly in its critical role in cancer diagnosis and treatment decision-making.

### 4.4 Directions for future research

Future research should prioritize the application of advanced AI techniques, including deep learning and CNNs, in the diagnosis and treatment of CRC. These technologies have the potential to automate the recognition and classification of CRC imaging data, significantly improving diagnostic accuracy and efficiency. For instance, new algorithms could be developed to autonomously detect subtle changes in early-stage tumors from radiological images. Additionally, generative adversarial networks are capable of generating high-quality synthetic medical images, which are invaluable in training AI models, especially in scenarios where data are scarce. This could enhance the models’ generalization abilities and increase the accuracy and reliability of their clinical applications. Combining multi-omics data (e.g., genomic, transcriptomic, proteomic, and metabolomic data) with radiomics is another vital research direction for advancing precision medicine. By integrating various levels of biological information, researchers can thoroughly analyze the biological characteristics of tumors and devise personalized treatment plans. Future studies might also leverage AI algorithms to investigate the correlation between gene expression and imaging features, potentially identifying novel biomarkers to predict treatment responses. Current technical challenges include data integration, the interpretability of algorithms, and the clinical translation of models. Despite the progress AI technology has made in radiomics, ensuring algorithmic transparency and interpretability remains crucial for clinician trust in AI decisions. Future technological advancements should aim to enhance the interpretive power of these algorithms and the safety of the systems to assure the reliability of AI applications in diverse clinical environments. The integration of AI with multi-omics data promises substantial improvements in CRC diagnosis and treatment. Through detailed analyses of the genetic characteristics of tumors and their imaging manifestations, it is feasible to accurately localize tumors, predict disease progression, and tailor treatment strategies, ultimately enhancing treatment outcomes.

### 4.5 Limitations of the study

This study employs bibliometric methods to evaluate the utilization of radiomics in CRC, shedding light on both the current trends and focal points in the field, as well as offering new perspectives for future research. However, bibliometric analysis inherently possesses certain limitations. Primarily, this method depends on published literature, which may not promptly reflect the latest research developments or technological innovations. Additionally, radiomics is a multidisciplinary field that intersects with medicine, biology, and computer science. The bibliometric approach might not capture the breadth of literature across all these relevant disciplines, potentially resulting in an incomplete analysis. To mitigate these limitations, future research should include multifaceted data from the literature by conducting systematic literature reviews or by integrating new theories and analytical tools, which would facilitate a more comprehensive analysis and enable the identification of more accurate research trends.

## 5 Conclusions

This study represents the first comprehensive bibliometric analysis of research in the field of radiomics in CRC since 2013. A systematic analysis of 1,226 publications from 2013 to 2023 was conducted to identify key contributors, institutions, and journals, as well as to examine the correlation between funding allocations and research focal areas. Through the integration of keyword and reference analysis, this research elucidates the current application of radiomics in CRC. It highlights its use in predicting molecular biomarkers, analyzing tumor aggressiveness, and assessing therapeutic responses. Future research should aim to further explore and integrate the multidisciplinary cross-application of innovative AI algorithms, multi-omics data (including genomics and proteomics), and imaging genomics. Such integration has the potential to enhance CRC prevention and the precision of medicine. This interdisciplinary approach is poised to not only improve the accuracy of disease diagnosis and treatment response prediction but also unveil new insights into the biological mechanisms of tumors and identify novel therapeutic targets. In conclusion, the application of radiomics in CRC management is evolving to be more diverse and in-depth. Its importance in the realm of precision medicine is anticipated to increase continually. It is hoped that more researchers will focus on this area to further advance the application of imagingomics in cancer prevention and treatment.

## Data Availability

The original contributions presented in the study are included in the article/**Supplementary Material**. Further inquiries can be directed to the corresponding authors.
