# Supplementary material for: Radiomics in precision medicine for colorectal cancer: a bibliometric analysis (2013–2023)
Source: Front Oncol. 2024 Oct 30;14:1464104. doi: 10.3389/fonc.2024.1464104 (PMC11571149; doi:10.3389/fonc.2024.1464104)
Supplement: Supplementary file 1 [file DataSheet1.docx]

**Supplementary Material: Cross-validation of Bibliometric Analysis Based on the Scopus Database**

1. **Introduction**

In the primary analysis, we conducted a bibliometric review of literature related to radiomics and colorectal cancer (CRC) using the Web of Science (WoS) Core Collection database. This review aimed to explore research trends and hotspots in the application of radiomics for CRC diagnosis, treatment, and prognosis. By employing bibliometric tools such as Bibliometrix, VOSviewer, and CiteSpace, we uncovered the significant role that artificial intelligence (AI) and medical imaging technologies have played in CRC research in recent years.

To further validate the robustness of our findings, we performed a cross-validation using the Scopus database, with the objective of confirming whether the research trends and hotspots identified in the WoS analysis align with those revealed by Scopus. As the Scopus database covers a broader range of publications and includes a larger volume of literature, it serves as a supplementary validation of the WoS findings. Additionally, the cross-validation using Scopus helps to enhance the generalizability of our conclusions and allows for an examination of differences in the scope and research focus between the two databases.

1. **Methods**

**2.1 Data Retrieval**

Relevant literature data were extracted from the Scopus database, covering the same time range as in the primary analysis, from January 1, 2013, to December 31, 2023.

The search query used was as follows:

(TITLE-ABS-KEY(("Colorectal Neoplasm*" OR "Colorectal Tumor*" OR "Colorectal Cancer*" OR "Colorectal Carcinoma*" OR "Colonic Neoplasm*" OR "Colon Neoplasm*" OR "Colon Cancer*" OR "Colonic Cancer*" OR "Cancer of the Colon" OR "Colon Adenocarcinoma*" OR "Rectal Neoplasm*" OR "Rectum Neoplasm*" OR "Rectal Tumor*" OR "Cancer of Rectum" OR "Rectal Cancer*" OR "Rectum Cancer*"))

AND TITLE-ABS-KEY(("radiomics" OR "image-based phenotyping" OR "imaging biomarkers" OR "texture analysis" OR "radiogenomics" OR "quantitative imaging" OR "imaging genomics" OR "image analysis" OR "computer-aided diagnosis" OR "machine learning in imaging")))

AND PUBYEAR > 2012 AND PUBYEAR < 2024 AND (LIMIT-TO(LANGUAGE, "English"))

This search was conducted on September 29, 2024, yielding a total of 2,790 articles and reviews.

**2.2 Data Processing and Analysis**

The data were preprocessed using Microsoft Excel 2019, where duplicate records were removed, and citation information for each publication was verified for completeness. VOSviewer and CiteSpace were employed to visualize and analyze the Scopus data, focusing primarily on publication trends and thematic hotspot analysis:

Publication trend analysis: The publication output in the Scopus dataset was statistically analyzed and compared to the annual publication trends from the WoS data. The consistency of research growth between the two databases was examined. Furthermore, the publication output of major contributing countries was analyzed, and a country collaboration network was constructed to illustrate the contributions and scientific collaboration in the field.

Thematic trend analysis: Reference clustering analysis was conducted, followed by the creation of a timeline chart to identify the core literature within the research field and track its evolution over time. Burst detection analysis was applied to identify key emerging references in recent years and analyze their time distribution to reveal newly developing research topics and areas of interest. Additionally, keyword clustering and timeline analysis were performed to visualize the temporal changes in various research topics, with a comparative analysis between Scopus and WoS data to confirm the consistency of research hotspots and thematic evolution.

1. **Results and Discussion**

**3.1 Descriptive Analysis**

The analysis based on Scopus data indicates that between 2013 and 2023, the annual publication volume in the field of CRC-related radiomics showed a consistent growth trend, which is highly aligned with the results from the Web of Science (WoS) data. Specifically, from 2013 to 2018, the publication volume exhibited slow growth, while from 2019 to 2021, there was a rapid increase in publications, particularly as AI and radiomics applications matured. In 2022 and 2023, the growth in publication volume slowed (see Figure 1A), which may be influenced by global research trends and economic factors. Although Scopus recorded a slightly higher total publication volume than WoS, mainly due to Scopus's broader coverage, while WoS applies stricter selection criteria, both databases show similar growth trends in key years, affirming the overall development trajectory of the field.

In terms of country contributions, the countries with the highest publication volumes in Scopus data are China and the United States, a finding that is fully consistent with WoS data (see Figure 1B). China leads in publication volume, whereas the United States continues to hold the lead in citation impact, demonstrating the significant global influence of U.S.-based research. A comparative analysis of the top 10 countries by publication volume and citations (see Table 1) reveals that both databases show similar contributions in international scientific collaboration, with only minor differences in country rankings. This consistency suggests that, despite the differences in journal coverage between the two databases, the major countries' contributions and influence in this field remain highly comparable.

The Scopus data's country collaboration network (see Figure 1C) reveals extensive collaboration between China, the United States, the United Kingdom, and other nations in CRC radiomics research, closely resembling the collaboration patterns observed in WoS data. The main collaboration networks remain concentrated in developed countries and institutions with abundant research resources, indicating stable global scientific collaborations. Timeline analysis (see Figure 1C) shows that international research collaboration has strengthened since 2015, particularly with emerging nations such as China and India, increasingly collaborating with the United States and European countries.

In the author collaboration network, Scopus data reveals a similar pattern to WoS data in identifying influential authors (see Figure 1D). However, there are some differences in publication and citation metrics. Specifically, Tong Tong is the most prolific author in the Scopus dataset, while Dekker Andre ranks first in citations (see Table 2). Although Tian J (Chinese Academy of Sciences) and Ganeshan B (University of London) are not the highest in publication or citation volume, they occupy crucial positions within the collaboration network, indicating their significant influence in scientific collaborations within this field.

These key nodes in the collaboration network demonstrate that while an author’s publication or citation volume may not be the highest, their broad collaborations are still driving progress in CRC radiomics research. Tian J and Ganeshan B have established wide-ranging collaborations with multiple researchers and institutions, further reinforcing their academic influence within the CRC radiomics field. This result aligns with findings from WoS data, highlighting the strong consistency across databases in identifying core contributors to this research area.

The top 10 journals in terms of publication volume and citation count in Scopus data align closely with the rankings observed in WoS data (see Table 3). *Frontiers in Oncology* and *Radiology* remain the leading journals in this field, showing a significant advantage in both publication output and citation impact. These two journals continue to drive research progress in CRC radiomics, underscoring their central influence in the field. The Scopus data further validates the findings from WoS, confirming that these high-impact journals hold a crucial position in CRC radiomics research.


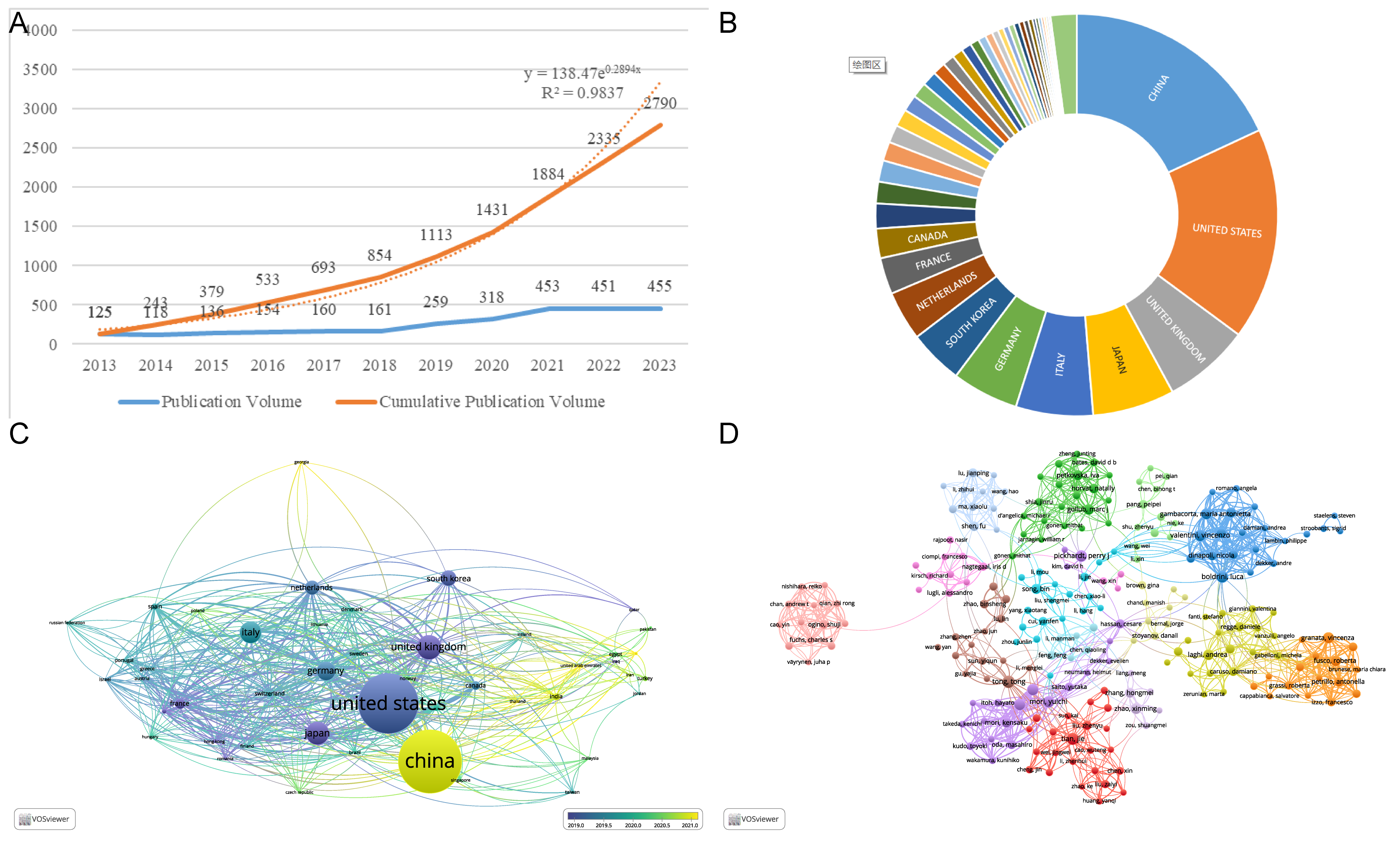


Figure 1. Analysis of CRC-related Radiomics Publications (2013-2023) Based on Scopus Data: (A) Annual publication trends in CRC-related radiomics research; (B) Country distribution of publication volumes; (C) International collaboration network over time; (D) Author collaboration network analysis.

Table 1: Top 10 Countries/Regions by Number of Publications

| Country | Count | Citation |
| --- | --- | --- |
| United States | 643 | 25407 |
| United Kingdom | 264 | 19917 |
| China | 680 | 16099 |
| Netherlands | 150 | 14171 |
| Germany | 201 | 13249 |
| Italy | 231 | 10739 |
| France | 111 | 10089 |
| Japan | 247 | 9462 |
| Spain | 65 | 6284 |
| Belgium | 53 | 5613 |

Table 2 Top Ten Most Prolific and Most Cited Authors

| Author | Count | Cited Author | Citation |
| --- | --- | --- | --- |
| Tong Tong | 20 | Dekker Andre | 4277 |
| Boldrini Luca | 19 | Lambin Philippe | 3729 |
| Tian Jie | 19 | Tian Jie | 3229 |
| Kudo Shin-ei | 18 | Sirinukunwattana Korsuk | 1929 |
| Mori Yuichi | 18 | Rajpoot Nasir M | 1904 |
| Valentini Vincenzo | 18 | Bernal Jorge | 1824 |
| Misawa Masashi | 17 | Chen Xin | 1638 |
| Pickhardt Perry J | 17 | He Lan | 1602 |
| Gambacorta Maria Antonietta | 16 | Liu Zhenyu | 1530 |
| Granata Vincenza | 16 | Chen Hao | 155 |

Table 3: Top 10 Journals by Number of Publications and Citations

| Journal | Count | JCR(2023) | IF(2023) | Cited Journal | Citation | JCR(2023) | IF(2023) |
| --- | --- | --- | --- | --- | --- | --- | --- |
| *Frontiers in Oncology* | 129 | Q2 | 3.5 | *Radiology* | 805 | Q1 | 12.1 |
| *Abdominal Radiology* | 77 | Q2 | 2.3 | *European Radiology* | 529 | Q1 | 4.7 |
| *Cancers* | 75 | Q1 | 4.5 | *Journal of Clinical Oncology* | 493 | Q1 | 42.1 |
| *European Radiology* | 75 | Q1 | 4.7 | *Lancet Oncology* | 385 | Q1 | 41.6 |
| *European Journal of Radiology* | 50 | Q1 | 3.2 | *New England Journal of Medicine* | 382 | Q1 | 96.2 |
| *Plos One* | 50 | Q1 | 2.9 | *Clinical Cancer Research* | 380 | Q1 | 10 |
| *World Journal of Gastroenterology* | 46 | Q1 | 4.3 | *Cancer Research* | 369 | Q1 | 12.5 |
| *Diagnostics* | 35 | Q1 | 3 | *Gastroenterology* | 369 | Q1 | 25.7 |
| *Academic Radiology* | 33 | Q1 | 3.8 | *Plos One* | 356 | Q1 | 2.9 |
| *Scientific Reports* | 31 | Q1 | 3.8 | *European Journal of Radiology* | 350 | Q1 | 3.2 |

- 1. **Hotspot and Trend Analysis**

Through reference clustering analysis of Scopus data, we identified key research areas in CRC, including texture analysis, external validation, artificial intelligence, colorectal cancer staging, resectable rectal cancer, predicting response, deep learning, and 3T MRI. These research hotspots are highly consistent with the findings from WoS data, confirming the importance of these topics in CRC radiomics research.

The reference timeline analysis reveals that, prior to 2015, research in the CRC field mainly focused on technologies like MRI and issues related to inter-software variability. However, after 2015, the research emphasis shifted toward specific clinical applications and diagnostic techniques such as metastasis, rectal neoplasms, colonoscopy, lymph nodes, response, and digital pathology. This trend reflects the evolution of the field from foundational technology research to clinical practice, and it aligns with the developmental path observed in WoS data.

The burst detection analysis further demonstrates that several key papers have emerged over the past five years, and these papers are frequently cited in both Scopus and WoS data. The burst references in Scopus are primarily centered on the applications of radiomics, deep learning, and artificial intelligence, with a focus on exploring the potential of these emerging technologies in CRC diagnosis and prognosis. These findings are in complete agreement with the research directions highlighted in WoS data.

The keyword clustering analysis of Scopus data shows a strong alignment with the results from WoS data regarding the primary research hotspots in CRC radiomics. Keywords such as colorectal liver metastases, colon cancer, artificial intelligence, rectal cancer, positron emission, metastatic colorectal cancer, preoperative prediction, and colonic lesion are central themes in the field. This validates the sustained focus on CRC, its associated technologies, and diseases in both databases.

The keyword timeline analysis of Scopus data indicates that, from 2015 to 2017, texture analysis and rectal cancer were the dominant research topics. Starting from 2018, deep learning and artificial intelligence emerged as core themes. This trend mirrors the research evolution in WoS data, highlighting the rapid growth of these technologies in medical image analysis and their increasing importance in CRC research. The consistency and synchronicity of research hotspots between the two databases underscore the global research trends in this field.


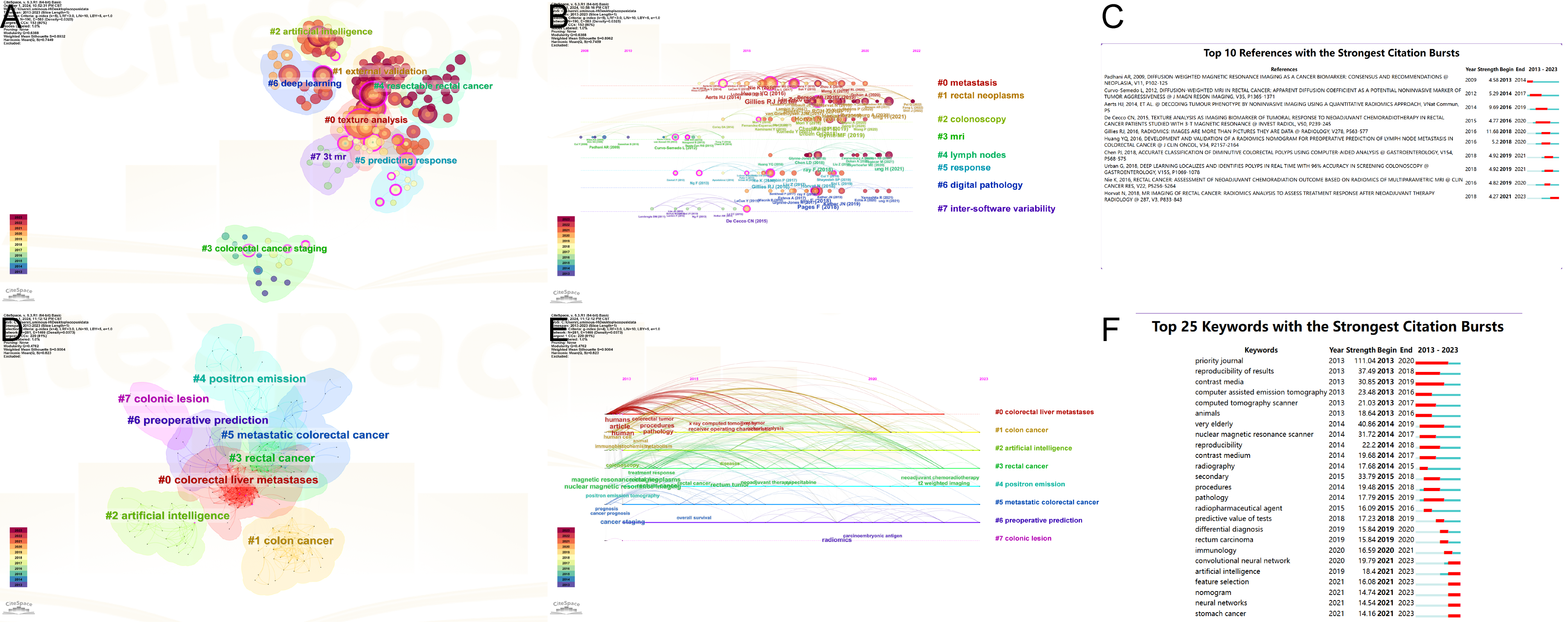


Figure 2. Thematic and Keyword Analysis of CRC-Related Radiomics Based on Scopus Data:

(A) Reference clustering analysis; (B) Reference timeline analysis; (C) Burst detection of key references; (D) Keyword clustering analysis; (E) Keyword timeline analysis; (F) Burst detection of top 25 keywords.

1. **Conclusion**

The cross-validation of Scopus and WoS data reveals a generally consistent trend in the keywords, research hotspots, and collaborative networks within the field of CRC radiomics. While the Scopus data provides broader coverage and emphasizes the importance of artificial intelligence (AI) and deep learning in this domain, the WoS data further highlights the trend toward multi-omics integration, such as the convergence of genomics with radiomics and AI. This trend was less evident in the Scopus data.

In the publication analysis, both Scopus and WoS data show a sustained growth in CRC radiomics research, peaking between 2019 and 2021. The primary countries contributing to publications and the patterns of international collaboration are consistent across both databases, underscoring the leading roles of China, the United States, and the enhancement of global scientific cooperation in this field.

However, in the thematic trend analysis, Scopus data tends to focus more on the application of AI and deep learning, whereas WoS data reflects a broader trend toward multi-omics integration, including the convergence of radiomics, genomics, and AI. This suggests that WoS provides a more comprehensive view of the multidimensional research directions within the field, while Scopus focuses more on specific technologies, such as AI and deep learning.

Nonetheless, burst detection analysis indicates that AI and deep learning are key research hotspots shared by both datasets and have emerged as dominant themes over the past three years. This confirms that these technologies will remain central to future CRC radiomics research.

In conclusion, while there are some differences in research trends between Scopus and WoS, particularly regarding multi-omics integration, both databases highlight the consistency of research hotspots related to AI and deep learning. This cross-database validation strongly supports the conclusions of the primary analysis. It also indicates that the global research directions in CRC radiomics remain highly aligned, and future studies should further emphasize the integration of multidisciplinary approaches, advancing radiomics for precision medicine.
